# Time spent in oxygen saturation 95–99% is associated with reduced mortality in critically ill patients with mechanical ventilation

**DOI:** 10.1186/s13054-020-03126-8

**Published:** 2020-07-09

**Authors:** Dawei Zhou, Zhimin Li, Guangzhi Shi, Jianxin Zhou

**Affiliations:** Department of Critical Care Medicine, Beijing Tiantan Hospital, Capital Medical University, Beijing, China

To the Editor:

The administration of supplemental oxygen is one of the ubiquitous interventions in the intensive care unit (ICU) and can be life-saving for mechanically ventilated patients [1]. However, excessive oxygen could be detrimental. Recently, several studies comparing the effect of conservative and liberal oxygen therapy for critically ill patients did not achieve consistent results [2, 3]. Furthermore, in patients with acute respiratory distress syndrome (ARDS), conservative oxygen therapy even had a signal of increased mortality and mesenteric ischemia [4]. Of note, the target oxygen levels in these studies were not the same. It is of paramount importance to elucidate oxygen targets to guide future research. In the present study, with a big database, we aimed to evaluate the association of the proportion of time within arterial oxygen saturation (SpO_2_) with hospital mortality in an ICU population with mechanical ventilation (MV).

This study used data stored in the eICU (eicu-crd.mit.edu) database [5]. Adult patients admitted to ICU for the first time with MV during the first 24 h were included. The main exposure was SpO_2_, which was generally interfaced from bedside vital sign monitors as the 5-min median value. Thirteen categories of SpO_2_ were generated, which were ≤ 88%, 89%, 90%, 91%, 92%, 93%, 94%, 95%, 96%, 97%, 98%, 99%, and 100%. The proportion of time spent (PTS) in different SpO_2_ categories for each patient was defined as the percentage of the summarized time in each different SpO_2_ category divided by total time. Thus, during the first 24 h, patients had SpO_2_ values that fell in the 13 categories and for each patient PTS in each of the predefined categories ranged from 0 to 100%. PTS was examined as both a continuous and categorical variable. The primary outcome was hospital mortality. Multivariable logistic regression models including PTS within each of these SpO_2_ categories along with the confounders were used to analyze the association of PTS-SpO_2_ with mortality outcome.

A total of 25,669 patients from 186 hospitals were included (Table 1), including 21,326 (83%) survivors and 4343 (17%) non-survivors. The median fraction of inspired oxygen was 45% (IQR, 43~60%) and the median duration of MV was 3 days (IQR, 2~5 days). After adjusted for confounders, PTS-SpO_2_ of ≤ 88%, 89%, 90%, 91%, 92%, 93%, and 100% were associated with a higher odds ratio for hospital mortality; PTS-SpO_2_ of 95%, 96%, 97%, 98%, and 99% were associated with a lower odds ratio; and PTS-SpO_2_ of 94% was not associated with hospital mortality (Fig. 1a). Based on the results, SpO_2_ was divided into three categories (≤ 94%, 95–99%, and 100%). PTS-SpO_2_ within categories of ≤ 94% (*p* < 0.001) and 100% (*p* < 0.001) were associated with a higher risk of hospital mortality, whereas an inverse trend was observed between PTS-SpO_2_ of 95–99% (*p* < 0.001) and hospital mortality (Fig. 1b).

**Table 1:** Characteristics of study patients between survivors and non-survivors

Variables	Total (*n* = 25, 669)	Survivors (*n* = 21, 326)	Non-survivors (*n* = 4343)	*p* value
Age, years (median, [IQR])	65 (54, 75)	65 (53, 74)	70 (58, 79)	< 0.001
Gender, male (*n* (%))	13,933 (54)	11,561 (54)	2372 (55)	0.636
BMI (median, [IQR])	28.3 (23.9, 34.4)	28.5 (24.1, 34.6)	27.4 (23.2, 33.1)	< 0.001
Comorbidities (*n* (%))				
Hypertension	13,533 (53)	11,216 (53)	2317 (53)	0.371
Diabetes mellitus	6149 (24)	5173 (24)	976 (22)	0.013
COPD	5870 (23)	4919 (23)	951 (22)	0.099
Heart failure	5011 (20)	4110 (19)	901 (21)	0.027
Cirrhosis	443 (2)	335 (2)	108 (2)	< 0.001
Cancer	411 (2)	269 (1)	142 (3)	< 0.001
Chronic renal failure	3585 (14)	2871 (13)	714 (16)	< 0.001
ICU types (*n* (%))				< 0.001
Med-Surg ICU	13,737 (54)	11,477 (54)	2260 (52)	
Cardiac ICU	1636 (6)	1216 (6)	420 (10)	
CCU-CTICU	2162 (8)	1843 (9)	319 (7)	
CSICU	889 (3)	768 (4)	121 (3)	
CTICU	1179 (5)	1083 (5)	96 (2)	
MICU	2587 (10)	2088 (10)	499 (11)	
Neuro ICU	1643 (6)	1295 (6)	348 (8)	
SICU	1836 (7)	1556 (7)	280 (6)	
Admission diagnosis (*n* (%))				< 0.001
Respiratory	5910 (23)	5106 (24)	804 (19)	
Sepsis	3660 (14)	2856 (13)	804 (19)	
Cardiac surgery	3035 (12)	2847 (14)	88 (2)	
Non-cardiac surgery	2495 (10)	2207 (10)	288 (7)	
Neurological	2560 (10)	1985 (9)	575 (13)	
Cardiovascular	2079 (8)	1783 (8)	296 (7)	
Cardiac arrest	2143 (8)	1149 (5)	994 (23)	
Trauma	1179 (5)	986 (5)	193 (4)	
Gastrointestinal	461 (2)	378 (2)	83 (2)	
Others	2147 (8)	1929 (9)	218 (5)	
TWM-PaO_2_, mmHg (*n* (%))				< 0.001
< 60	622 (2)	480 (2)	142 (3)	
60–120	10,593 (41)	8832 (41)	1761 (41)	
120–300	8226 (32)	6691 (31)	1535 (35)	
> 300	579 (2)	453 (2)	126 (3)	
Missing (*n* (%))	5649 (22)	4870 (23)	779 (18)	
TWM-PaCO_2_, mmHg (*n* (%))				< 0.001
< 35	4420 (17)	3336 (16)	1084 (25)	
35–45	9555 (37)	8041 (38)	1514 (35)	
> 45	5849 (23)	4928 (23)	921 (21)	
Missing (*n* (%))	5845 (23)	5021 (24)	824 (19)	
TWM-pH (*n* (%))				< 0.001
< 7.35	6868 (27)	5311 (25)	1557 (36)	
7.35–7.45	10,085 (39)	8635 (40)	1450 (33)	
> 7.45	2626 (10)	2136 (10)	490 (11)	
Missing (*n* (%))	6090 (24)	5244 (25)	846 (19)	
TWM-FiO_2_, % (median, [IQR])	45 (43, 60)	45 (42, 59)	50 (45, 75)	< 0.001
APACHE IV (median, [IQR])	68 (50, 89)	63 (48, 83)	92 (72, 115)	< 0.001
SOFA (median, [IQR])	6 (4, 8)	6 (4, 8)	8 (6, 11)	< 0.001
Vasopressors (*n* (%))	5734 (22)	4135 (19)	1599 (37)	< 0.001
Dialysis (*n* (%))	976 (4)	802 (4)	174 (4)	0.466
Ventilation days (*n* (%))	3 (2, 5)	3 (2, 4)	4 (2, 7)	< 0.001

*IQR* interquartile range, *BMI* body mass index, *COPD* chronic obstructive pulmonary disease, *ICU* intensive care unit, *CCU* coronary care unit, *CTICU* cardiothoracic ICU, *CSICU* cardiac surgery ICU, *MICU* medical ICU, *SICU* surgical ICU, *TWM* time-weighted mean, *SpO*_*2*_ peripheral oxygen saturation, *PaO*_*2*_ partial pressure of arterial oxygen, *PaCO*_*2*_ partial pressure of arterial carbon dioxide, *FiO*_*2*_ fraction of inspired oxygen, *APACHE* Acute Physiology and Chronic Health Evaluation, *SOFA* sequential organ failure assessment

**Fig. 1:**
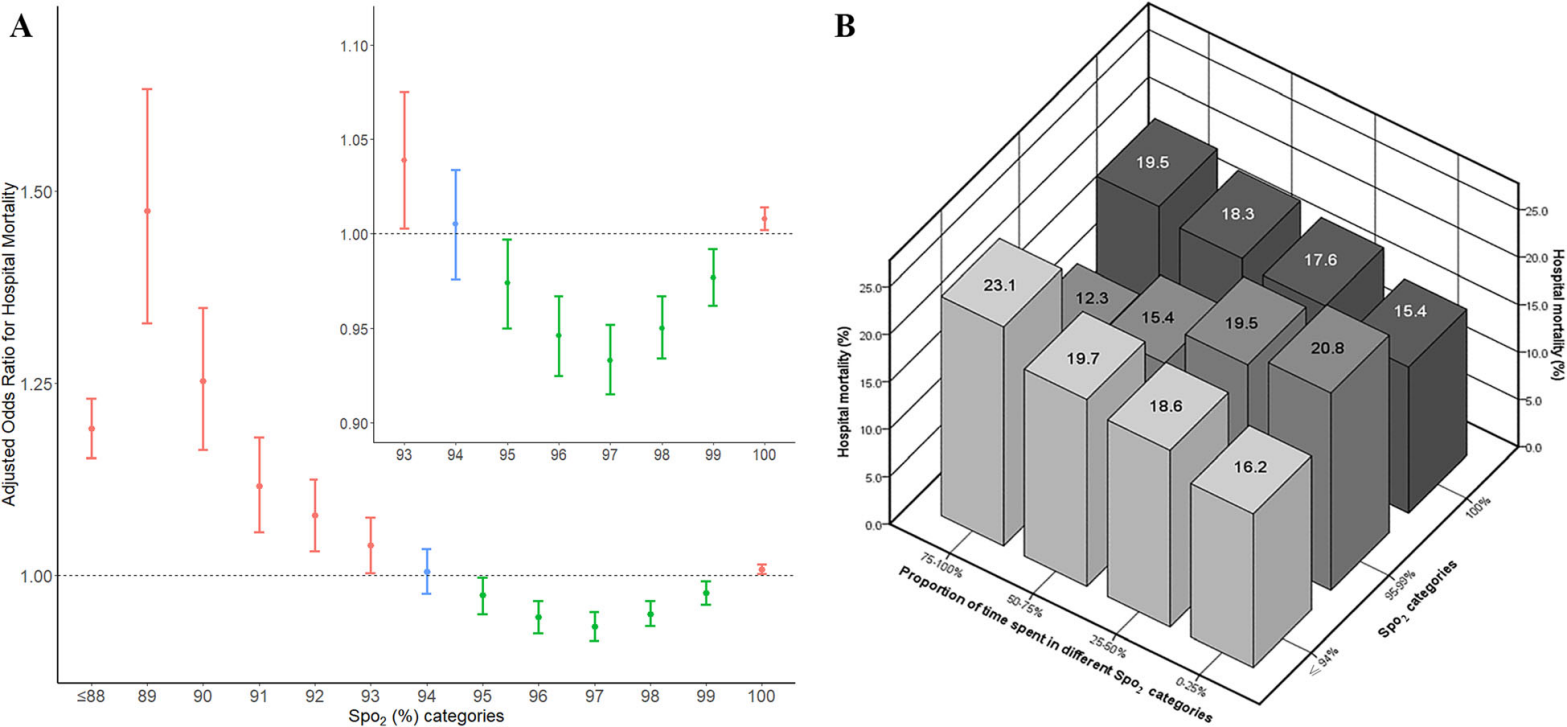
**a** Adjusted odds ratio for hospital mortality according to different SpO_2_ categories. The proportion of time spent in thirteen different SpO_2_ categories (≤ 88%, 89%, 90%, 91%, 92%, 93%, 94%, 95%, 96%, 97%, 98%, 99%, and 100%) was considered as a continuous variable, ranging from 0 to 100%, and was included in separate multivariable regression models along with the confounders. In total, 13 models were created. The adjusted odds ratio for each SpO_2_ category and 95% confidence intervals (error bars) were calculated after adjusting for age, body mass index (obesity or non-obesity), admission diagnosis, comorbidities (diabetes mellitus, cancer), time-weighted mean FiO_2_, time-weighted mean pH, time-weighted mean PaCO_2_, sequential organ failure assessment score (not including the respiratory part), and use of dialysis. An odds ratio is calculated per 5% increase in time in each given category. SpO_2_, arterial oxygen saturation; FiO_2_, fraction of inspired oxygen; PaCO_2_, partial pressure of arterial carbon dioxide. **b** Observed hospital mortality of four predefined time ranges (0–25%, 25–50%, 50–75%, and 75–100%) spent in three different SpO_2_ categories (≤ 94%, 95–99%, and 100%). Figures on each histogram column represented the crude hospital mortality

The result of the present study was partially consistent with the British Thoracic Society guideline, which recommended the target of SpO_2_ 94–98% [6]. In addition, the result could partly account for the discrepancy of the recent clinical trials of oxygen therapy, which adopted different target oxygen levels [2–4]. Despite several limitations to our study (e.g., retrospective design, potential residual confounders, unvalidated data from monitors, relatively short study period, lack of mode of MV, and missing data), our study provided observational evidence for a SpO_2_ target range of 95–99% with real-world data. Further studies are warranted to validate the particular target.

In conclusion, the proportion of time spent in oxygen saturation 95–99% is associated with reduced mortality in critically ill patients with mechanical ventilation. These findings may have implications for the design of future trials of oxygen therapy.

## Data Availability

Data analyzed during the present study are currently stored in the eICU database (eicu-crd.mit.edu). After completing the required training course (the Collaborative Institutional Training Initiative) and requesting access to the eICU Collaborative Research Database, researchers can seek to use the database.

## Abbreviations

APACHE: Acute Physiology and Chronic Health Evaluation
ARDS: Acute respiratory distress syndrome
FiO_2_: Fraction of inspired oxygen
ICU: Intensive care unit
IQR: Interquartile range
MV: Mechanical ventilation
OR: Odds ratio
PaO_2_: Partial pressure of oxygen
PaCO_2_: Partial pressure of arterial carbon dioxide
PTS-SpO_2_: Proportion of time spent in SpO_2_
SOFA: Sequential organ failure assessment
SpO_2_: Arterial oxygen saturation
TWM: Time-weighted mean
